# Supplementary material for: RNAseq-Based Carboxylesterase Nl-EST1 Gene Expression Plasticity Identification and Its Potential Involvement in Fenobucarb Resistance in the Brown Planthopper Nilaparvata lugens
Source: Insects. 2024 Sep 26;15(10):743. doi: 10.3390/insects15100743 (PMC11508480; doi:10.3390/insects15100743)
Supplement: Supplementary file 1 [file insects-15-00743-s001.zip › insects-3179277-supplementary.pdf]

**Table S1**. Raw data statistics of RNAseq

| Index | Sample id | Total read bases* | Total reads | GC (%) | Q30 (%) |
|---|---|---|---|---|---|
| 1 | BPH80-1 | 6,029,785,446 | 59,700,846 | 39 | 95 |
| 2 | BPH80-2 | 5,562,620,248 | 55,075,448 | 39 | 95 |
| 3 | BPH80-3 | 5,600,790,976 | 55,453,376 | 40 | 95 |
| 4 | BPH15-1 | 6,151,718,100 | 60,908,100 | 33 | 95 |
| 5 | BPH15-2 | 6,468,012,326 | 64,039,726 | 31 | 95 |
| 6 | BPH15-3 | 5,391,991,050 | 53,386,050 | 32 | 95 |
| 7 | BPH19-1 | 5,827,667,680 | 57,699,680 | 37 | 96 |
| 8 | BPH19-2 | 6,091,218,898 | 60,309,098 | 37 | 95 |
| 9 | BPH19-3 | 6,275,603,488 | 62,134,688 | 36 | 96 |

**Table S2**. Trimming data statistics of RNAseq

| Index | Sample id | Total read bases* | Total reads | GC (%) | Q30 (%) |
|---|---|---|---|---|---|
| 1 | BPH80-1 | 5,860,975,913 | 58,425,794 | 39 | 96 |
| 2 | BPH80-2 | 5,419,295,619 | 53,959,610 | 39 | 96 |
| 3 | BPH80-3 | 5,410,374,591 | 53,922,930 | 40 | 96 |
| 4 | BPH15-1 | 6,002,133,878 | 59,835,472 | 33 | 96 |
| 5 | BPH15-2 | 6,337,003,490 | 63,107,390 | 31 | 96 |
| 6 | BPH15-3 | 5,263,870,871 | 52,423,690 | 32 | 96 |
| 7 | BPH19-1 | 5,708,437,661 | 56,812,832 | 37 | 96 |
| 8 | BPH19-2 | 5,958,250,055 | 59,327,242 | 37 | 96 |
| 9 | BPH19-3 | 6,146,600,633 | 61,249,890 | 36 | 96 |

· Total read bases: Total number of read bases after trimming
· Total reads: Total number of reads after trimming
· GC (%): GC Content
· Q30 (%): Ratio of bases that have phred quality score greater than or equal to 30

**Table S3**. Statistics of initial assembled contig

| Assembly | No of genes | No of transcripts | GC (%) | N50 | Avg. contig length (bp) | Total assembled bases (bp) |
|---|---|---|---|---|---|---|
| merge | 148,234 | 191,287 | 41 | 912 | 626 | 119,808,296 |
| BPH80 | 92,362 | 115,494 | 39 | 810 | 584 | 67,502,190 |
| BPH15 | 79,691 | 97,626 | 38 | 793 | 577 | 56,350,010 |
| BPH19 | 111,118 | 139,372 | 41 | 893 | 618 | 86,124,462 |

**Table S4**. Overall mapping ratio in the reference unigene set (merged) for each sample

| Sample | Number of processed reads | Number of mapped reads | Number of unmapped reads |
|---|---|---|---|
| BPH80-1 | 58,425,794 | 43,830,936 (75.02%) | 14,594,858 (24.98%) |
| BPH80-2 | 53,959,610 | 41,350,506 (76.63%) | 12,609,104 (23.37%) |
| BPH80-3 | 53,922,930 | 40,138,432 (74.44%) | 13,784,498 (25.56%) |
| BPH15-1 | 59,835,472 | 49,769,630 (83.18%) | 10,065,842 (16.82%) |
| BPH15-2 | 63,107,390 | 53,916,942 (85.44%) | 9,190,448 (14.56%) |
| BPH15-3 | 52,423,690 | 44,043,944 (84.02%) | 8,379,746 (15.98%) |
| BPH19-1 | 56,812,832 | 43,762,216 (77.03%) | 13,050,616 (22.97%) |
| BPH19-2 | 59,327,242 | 45,969,522 (77.48%) | 13,357,720 (22.52%) |
| BPH19-3 | 61,249,890 | 48,080,464 (78.5%) | 13,169,426 (21.5%) |

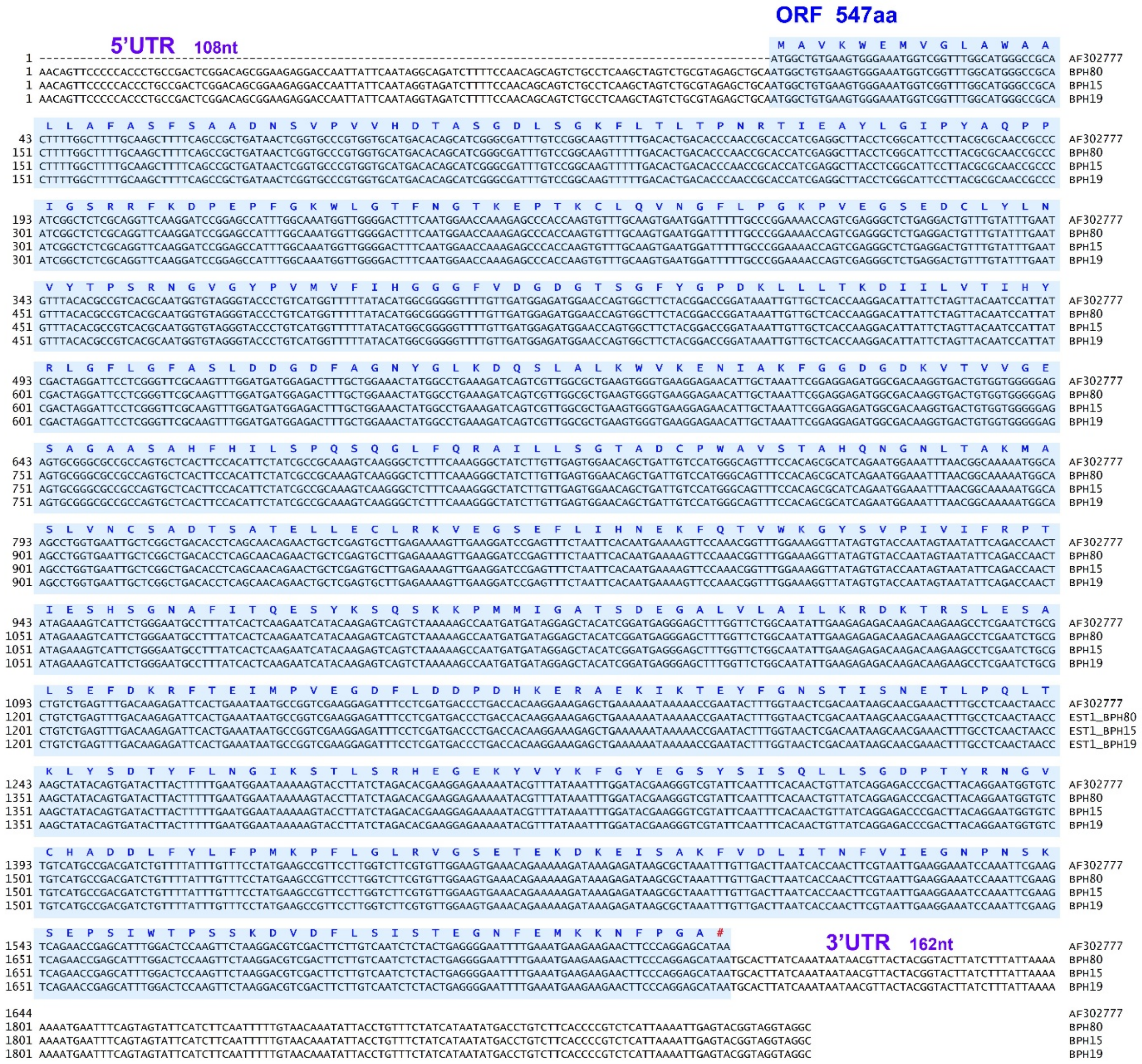


**Figure S1**. Comparative analysis of the *Nl-EST1* among all BPH strains at the cDNA level. No mutations were found compared to the previously known et al. (2012b) Nl-EST1 (AF30277) based on 547 amino acids. The blue letter indicates the amino acid sequence and the blue box indicates the ORF region. The 5'UTR of 108 nt and the 3'UTR of 162 nt were also compared.

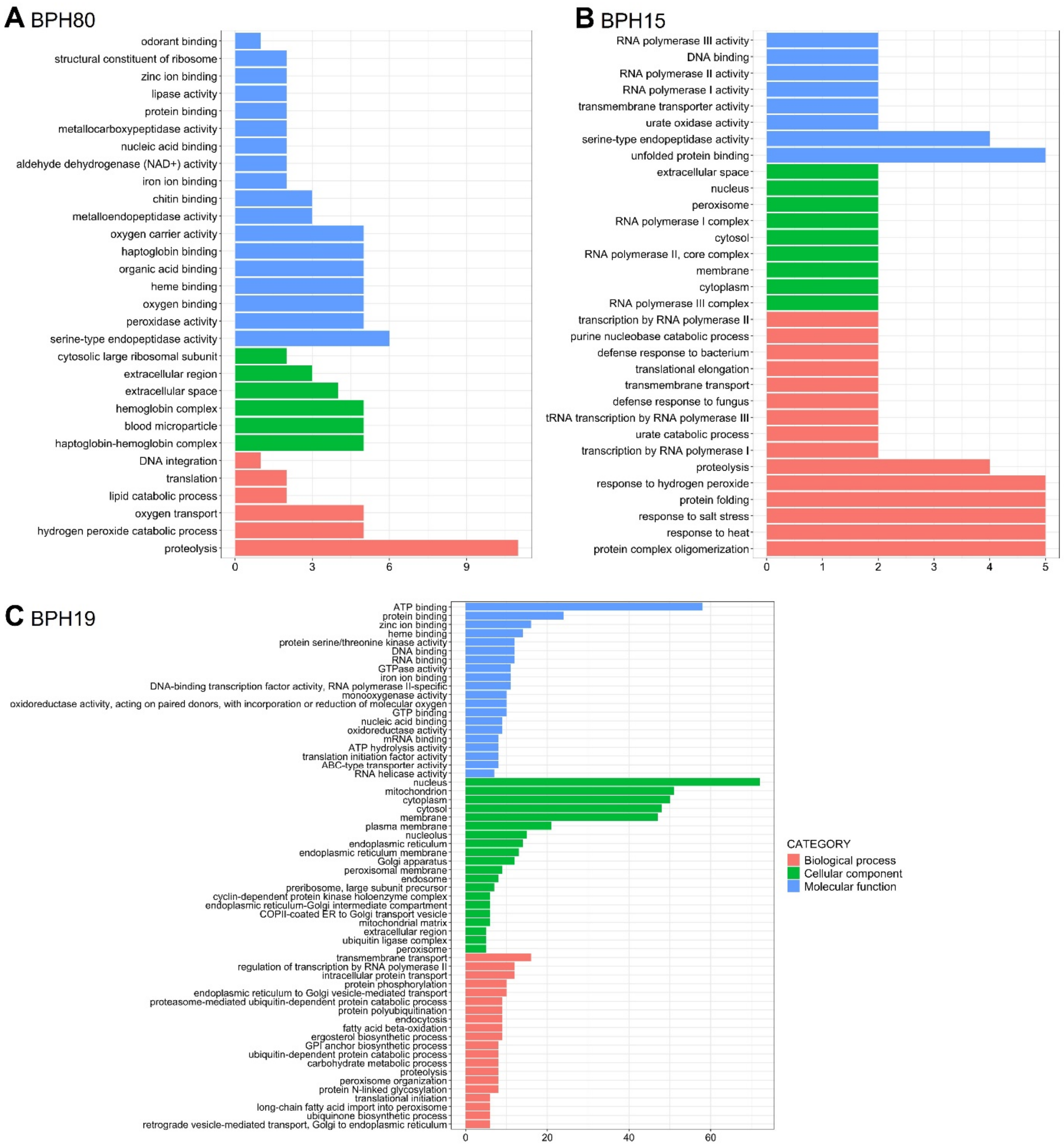


**Figure S2**. Gene ontology (GO) analysis results of all BPH strains specifically expressed genes. GO was performed on 20 genes of BPH80, 21 genes of BPH15, and 454 genes of BPH19 expressed in a strain or population-specific manner in Fig. 3C.

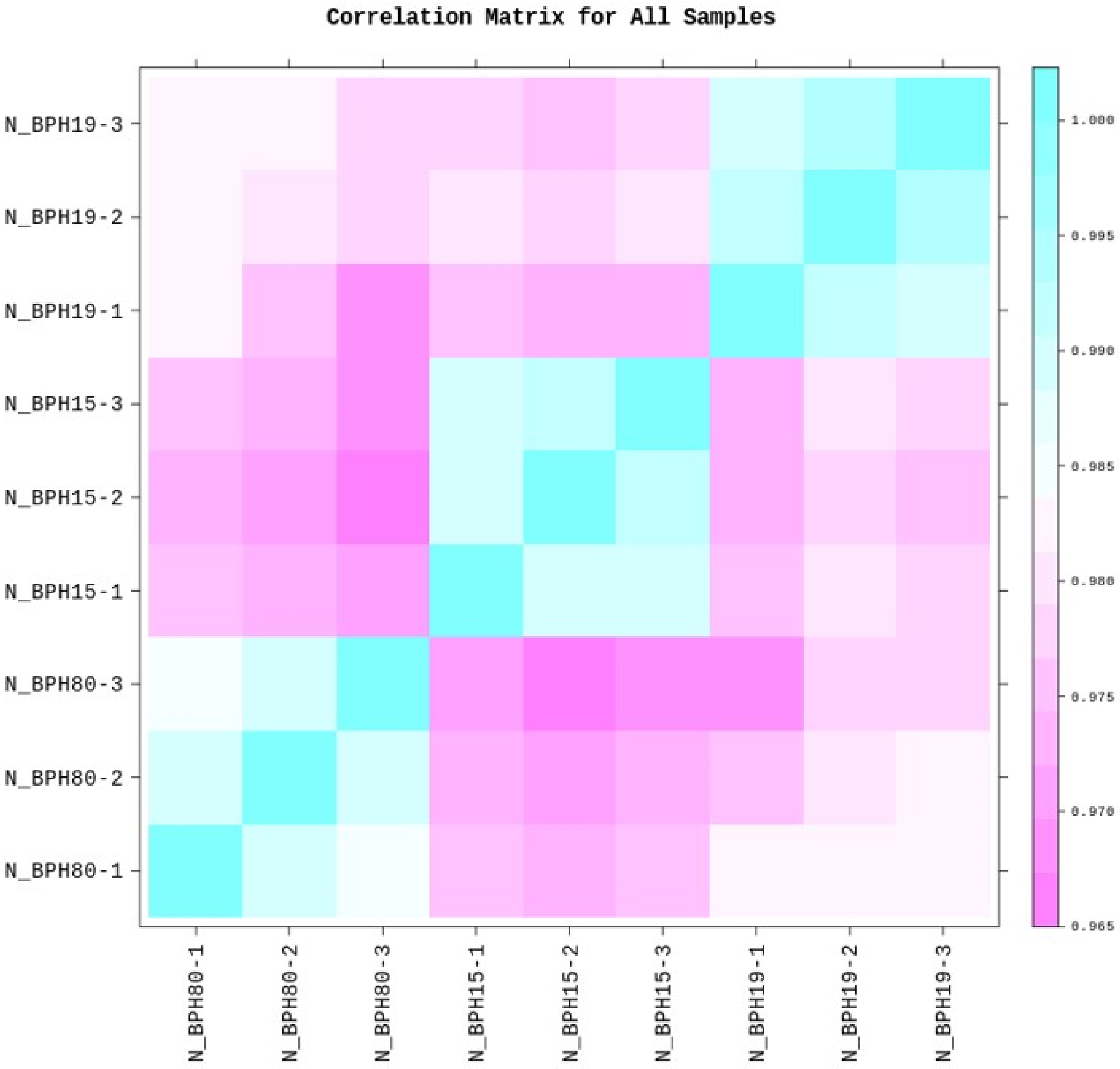


**Figure S3**. Correlation matrix analysis for all BPH samples.
